# Topolnogical classifier for detecting the emergence of epileptic seizures

**DOI:** 10.1186/s13104-018-3482-7

**Published:** 2018-06-14

**Authors:** Marco Piangerelli, Matteo Rucco, Luca Tesei, Emanuela Merelli

**Affiliations:** 1 Computer Science Division, School of Science and Technology, University of Camerino, Via Madonna delle Carceri, 9, 62032 Camerino, IT Italy; 2 ALES S.r.l.-United Technologies Research Center, Via Praga, 5, 38121 Trento, IT Italy

**Keywords:** Complex systems, Brain, Epilepsy, Topological data analysis, Persistent entropy, Time series

## Abstract

**Objective:**

An innovative method based on topological data analysis is introduced for classifying EEG recordings of patients affected by epilepsy. We construct a topological space from a collection of EEGs signals using Persistent Homology; then, we analyse the space by Persistent entropy, a global topological feature, in order to classify healthy and epileptic signals.

**Results:**

The performance of the resulting one-feature-based linear topological classifier is tested by analysing the Physionet dataset. The quality of classification is evaluated in terms of the Area Under Curve (AUC) of the receiver operating characteristic curve. It is shown that the linear topological classifier has an AUC equal to $97.2\%$ while the performance of a classifier based on Sample Entropy has an AUC equal to 62.0%.

## Introduction

Epilepsy is a chronic brain disorder characterised by recurrent seizures of several entity with different manifestations. They are caused by sudden excessive electrical discharges in a group of neurons [1] and they are defined as a spontaneous hyper-synchronous activity of clusters of neurons [2].

Human brain can be considered as a complex self-adaptive system composed of billions of non-identical neurons, entangled in loops of non-linear interactions, determining the brain behaviours [3]. Epilepsy is just an example of such behaviours: identifying the onset of a neural hyper-synchronisation is similar to discovering patterns of information expressed by a network of interactions in the space of neurons.

The electroencephalogram (EEG) is the standard technique used to record the electrical activity of the brain. The *direct observation* of EEG signals helps neurologists in diagnosing epilepsy while *automatic methods* for this task are still not used even if, in the last decades, several methods for automatic diagnosis have been proposed in the literature [4–8]. The intrinsic non-linearity and non-stationarity of EEG signals requires methods capable of extracting *global* information, characterising the processes described by the signals.

Topological data analysis (TDA) is able to extract such information [9–14]; currently, it has been used for the analysis of EEG signals [15] within the TOPDRIM project [16]. The key-concept in TDA is *persistent homology*: a procedure for counting, through a process called *filtration*, the higher dimensional persistent holes of topological spaces. Its visualisation can be given as persistent barcodes or as persistent diagrams.

In this paper we describe the realisation of a Persistent Entropy-based classifier to discriminate the epileptic EEG signals from the non-epileptic ones. The proposed method defines an automatic classifier of signals and it is a preliminary step towards the study of an automatic detection of epileptic seizures. Afterwards, we use the Vietoris–Rips filtration for understanding how the regions of the brain are involved in the spreading of epileptic signals.

## Main text

### Material and methods

#### Dataset

**Fig. 1:**
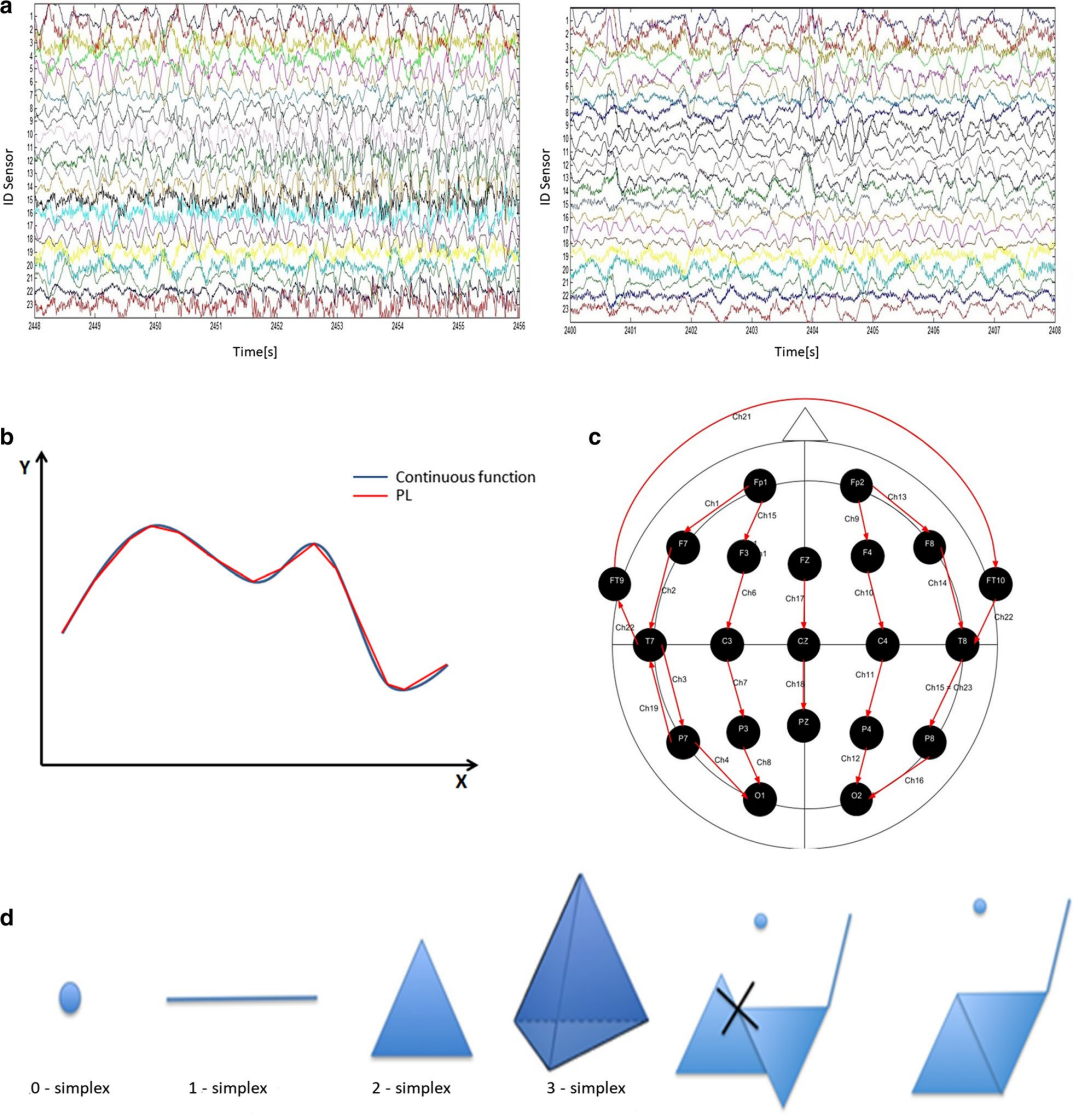
**a** Examples of epileptic (on the left) and healthy (on the right) EEG recordings. The amplitude of each signal is in $\mu$V. **b** An example of a PL. **c** Graphical scheme representing the positions of the electrodes during an EEG. The arrows correspond to the 23 potential differences that are recorded. **d** Geometrical representation of some simplices, followed by an aggregation of simplices that is not a simplicial complex because the intersection of the two triangles is not a face of any of them. The last aggregation is a proper simplicial complex

The dataset used consists of EEG signals, i.e. multivariate time series (see Fig. 1a), taken from the *PhysioNet* database [17]. EEGs are performed positioning electrodes at some key points on the patient’s head following some schemes: the database we used adopts the international 10–20 system (see Fig. 1c). The EEGs used in this study were collected at the Children’s Hospital Boston and they consist of recordings from pediatric subjects with intractable seizures. Subjects were monitored for several days following the withdrawal of anti-seizure medication in order to characterise their seizures and assess their candidacy for surgery. We selected 33 recordings with, at least, one epileptic event and 33 without epileptic events. The recordings have the same number of channels (electrodes), 23, with the same length, 921,600 samples with a sampling frequency of 256 Hz.

**Fig. 2:**
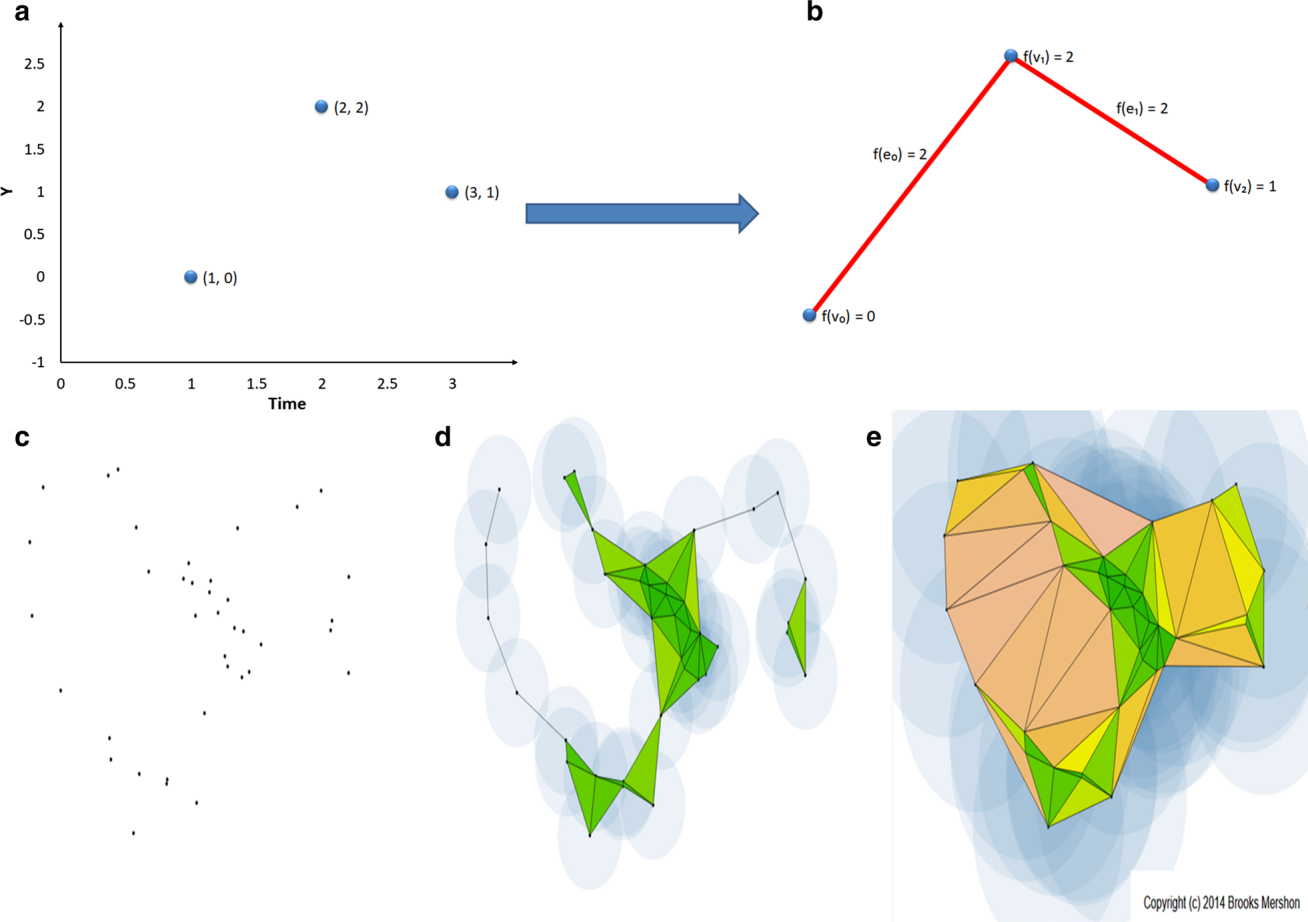
Graphical representation of the algorithms for the Piecewise and the Vitetoris-Rips filtrations. **a** The input signal, formed by three time points with coordinates (1, 0),  (2, 2) and (3, 1) respectively. **b** The filtered simplicial complex formed by three $0-$simplices: $\{v_0,v_1,v_2\}$ with filter values $f(v_0)=0, f(v_2)=1, f(v_1)=2$ and two $1-$simplices: $\{e_0, e_1\}$, with filter values $f(e_0)=f(e_1)=2$, so the set of filter values is $F = \{0,1,2\}$. **c** A PCD in a metric space. **d** Each point is surrounded with a sphere of radius *r* / 2 such that all the spheres grow up simultaneously and equally. The choice of the parameter *r* gives rise to certain pairwise intersections of the spheres, which determine the simplices forming the simplicial complex at filtration time *r*. A pairwise non-empty intersection of dimension *k* is equal to a $k-1$-simplex. **e** A sequence of increasing values for the parameter *r* gives rise to a filtration and a final simplicial complex *K* is formed with the maximum value of *r*. The Vietoris–Rips filtration is simply obtained by considering a sequence of increasing values of the parameter *r*. **c**, **d** and **e** are generated using the software by Brooks Mershon [35]

**Fig. 3:**
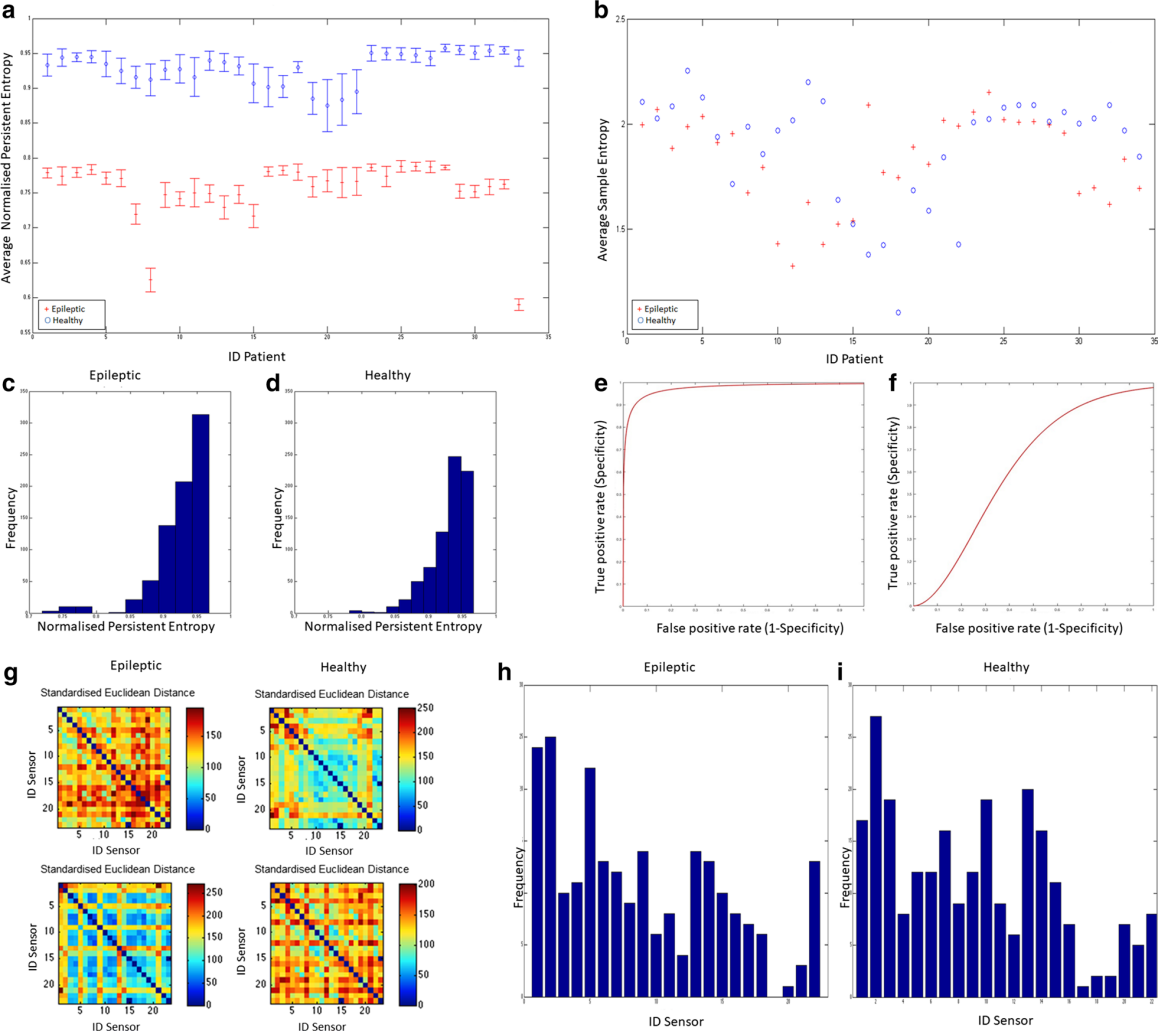
**a** $\widehat{\mathcal {H}^j}$ for epileptic (red) and healthy (blue) signals. **b** Average value of Sample Entropy over the 23 EEG signals of epileptic patients (red) and of healthy patients (blue). **c**, **d** Histogram of the frequencies of ${\mathcal {H}^j}$ values for each channel, for each patient and for the two classes of patients. The elements are sorted into 10 equally spaced bins along the x-axis between the minimum and maximum values of ${\mathcal {H}^j}$. **e** The ROC curve of the $\widehat{\mathcal {H}^j}$-based classifier (AUC = $97.2\%$). **f** The ROC curve of the Sample Entropy-based classifier (AUC = $62\%$). **g** Mosaic Plots representing the distance matrices of the recordings of four patients. The entries of the matrices are the pairwise Standardised Euclidean distances among sensors. The matrices are used in the calculation of Vietoris–Rips filtration. **h**, **i** Frequency of the generators, i.e. sensors, belonging to the found i-dimensional holes for 33 epileptic signals (left) and 33 healthy signals (right)

#### TDA: a new method for data analysis

Consider a set of points *G*, i.e. our data, embedded in a *d*-dimensional space $\mathbb {D}^d$ and assume that those data were sampled from an unknown *k*-dimensional space $\mathbb {D}^k$ with $k \le d$. Our task is to reconstruct the space $\mathbb {D}^k$ from the dataset *G*.

In TDA, G elements are equipped with a notion of *proximity* that characterises a coordinate-free metric. Those points are converted into topological spaces called *simplicial complexes*. Simplicial complexes are made up by building blocks called simplices: points are 0-simplices, line segments are 1-simplices, filled triangles are 2-simplices, filled tetrahedra are 3-simplices and so on (see Fig. 1d).

A *Filtration* is a collection of nested simplicial complexes. Building a filtration can be seen as wearing lenses for examining the dataset: different lenses consent to extract different kinds of information from the topological space. In this paper we use *Piecewise filtration* and *Vietoris–Rips filtration*. Choosing a filtration is a crucial step: different filtrations give rise to different conversions of the data points G into simplicial complexes [18–20].

##### *Piecewise filtration*

Piecewise filtration, recently introduced by Rucco et al. [21], is used for studying signals. The procedure is based on the well known concept of Piecewise Linear function (PL), $PL:\mathbb {R}\rightarrow \mathbb {R}$, shown in Fig. 2a, b.

##### *Vietoris–Rips filtration*

Vietoris–Rips filtration is used for studying Point Cloud Data (PCD). It creates a sequence of simplices, built on a metric space, used to add topological structure to an otherwise disconnected set of points [22, Chapter III]. Figure 2c, d, e show a graphical representation of this approach.

##### *Persistent homology*

Persistent homology is the combinatorial counterpart of *Homology*, an algebraic object that counts the number of *n*-dimensional holes in a topological space, the so-called Betti numbers. The filtration process is necessary for the computation of persistent homology. The set of Betti numbers is composed by $\beta _0$, the number of connected components in a generic topological space *K*; $\beta _1$, the number of holes in *K*; $\beta _2$, the number of voids in *K* and so on. Along the filtration, persistent homology calculates $k-$dimensional Betti intervals: a $k-$dimensional Betti interval $[t_{start}, t_{end}]$ defines the time at which a *k*-dimensional hole appears in the simplicial complex ($t_{start}$), while $t_{end}$ is the time at which it disappears. The holes that are still present at $t_{end}= t_{max}$ correspond to *persistent topological features* [23]. A graphical representation of those intervals in *K* is called *persistence barcode* and it is associated to a filtration. An equivalent representation is a *persistence diagram* [24]. An additional information returned by the computation of persistent homology is the list of the *generators*, which are the simplices involved in the holes. Experimentally, the generators play a crucial role for the description of the data under analysis [25, 26].

##### *Persistent entropy*

A new entropy measure called *Persistent entropy* has been recently introduced for measuring how much the construction of a filtered simplicial complex is “ordered” [27]. Given a topological space *K* and a set of the filtration parameters *F*, let $B = \{[x_i , y_i) \mid i \in I\}$, where *i* is a set of indexes, be the persistent barcode associated to the filtration of *K*. The Persistent entropy *H* of the filtered simplicial complex is calculated as follows:

$$\begin{aligned} H=-\sum _{i \in I} p_i \log {(p_i)} \end{aligned}$$

where $$p_i=\frac{\ell _i}{L}$$, $$\ell _i=y_i - x_i$$, and $$L=\sum _{i\in I}\ell _i$$. In case of a persistent interval $$[x_i~,~\infty )$$, an interval $$[x_i~,~m)$$ is used, where $$m = \max \{F\} + 1$$. Moreover, to rescale *H* in the interval [0, 1] and to compare the values from different barcodes we use the stability theorem for *H* and the normalised *H*, denoted by $$\mathcal {H}$$, and defined as:

$$\begin{aligned} \mathcal {H} = \frac{H}{\log {\ell _{\rm max}}} \end{aligned}$$

where $\ell _{\rm max}$ is the maximum interval length in the considered barcode [21].

### A new topological classifier for epilepsy

Given the above theoretical framework, let us define a new methodology for the analysis of EEG signals. It can be divided in three steps:

- *Step I* preprocessing of the input.
- *Step II* computation of *H* using the Piecewise filtration and derivation of a linear topological classifier (LTC).
- *Step III* identification of regions involved in the spreading of the epileptic signals using Vietoris–Rips filtration.

#### Step I

Let $j \in \{1, 2, \ldots , 66\}$ be the index of the EEG recordings, denoted by $\mathbb {S}^j$. Each $\mathbb {S}^j$ is composed of 23 one-dimensional signals, $\mathbb {S}^j=\{S_1^j, S_2^j, \ldots , S_{23}^j\}$, and each $S_i^j \subset \mathbb {R}^2$ is a PL function. The length of each $S_i^j$ is *N*, the number of samples. For each $S_i^j$ the preprocessing performs two actions:

1. *Filtering* the EEG reduces the noise by using a bandpass filter between 1–70 Hz, and removes the power line using a notch filter, between 8 and 52 Hz [28–30].
2. *Downsampling* the EEG reduces the time needed for the computation of the topological features during the subsequent steps. The worst-case complexity of computing persistent homology using the JavaPlex tool [31] is cubic in the number of simplices. This number is linear with respect to the number of points in case of piecewise complexes. Downsampling should be used if and only if it preserves the main geometrical characteristics of the original signals, that is the shape. In MATLAB we used the command “decimate” [32].

After the preprocessing, the signals were denoted $\tilde{S}_i^j$.

#### Step II

After performing the Piecewise filtration, we computed $\mathcal {H}$ for each $\mathbb {\tilde{S}}^j$ thus obtaining a vector of 23 values of $\mathcal {H}$. Then, we calculated the average value of this vector, $\widehat{\mathcal {H}^j}$. $\widehat{\mathcal {H}^j}$ is our 1-dimensional feature able to differentiate signals by looking at their shapes [21].

We repeated the procedure using *Sample Entropy*, a well-established technique in time series analysis [33, 34], on the same dataset. Finally, we trained an $\mathcal {H}$-based supervised classifier and a Sample Entropy-based supervised LTC. We randomly divided the dataset into a training ($70\%$) and a testing ($30\%$) subset. We applied a 10-fold cross validation.

#### Step III

Let us consider our dataset as a PCD: each patient is represented by 23 points in $\mathbb {R}^{N}$. Assuming that the generators of the persistent holes correspond to the sensors on the head of the patient, we applied the Vietoris–Rips filtration to determine which particular sensors (thus, which areas of the brain) are more “involved”$\backslash$“significant” concerning the spreading of epileptic seizures. Standardised Euclidean distance among sensors, see Fig. 3g, is the metric upon which we performed the Vietoris–Rips filtration. This metric is useful when the dataset contains heterogeneous scale variables and it is defined as:

$$\begin{aligned} d(\tilde{S},\tilde{S}')= \sqrt{ \sum _{k=1}^N \Big ( \frac{\tilde{S}|_k}{s_k} - \frac{\tilde{S}'|_k}{s_k} \Big )^2} \end{aligned}$$

where $\tilde{S}|_k$ stands for the *y*-component in $(x_k,y_k)$ of the channel signal $\tilde{S}$ and $s_k$ is the sample standard deviation calculated among the 23 *y*-components at position *k* of the signal $\tilde{\mathbb {S}}$ to which $\tilde{S}$ belongs.

## Results

We report the results of the analysis on the signals decimated by a factor 10, which produced 92160 samples per signal (*N* = 92160). We tested our method using the non-downsampling signals and using different values of the decimation factor ($df = 10$ and $df=100$). We report the results of the analysis using $df =10$ (because *H* did not show significative changes for $df= 100$). In Fig. 3c, d the frequency of the values of $\mathcal {H}_i^j$ is reported. The class of epileptic patients is characterised by a peak of 313 elements in the range [0.942, 0.967] of $\mathcal {H}$ values, with centre value 0.955. The class of healthy patients is characterised by a peak containing 247 elements with $\mathcal {H}$ values in [0.930, 0.948] with centre value 0.939. A strong separation between the two classes is clearly depicted in Fig. 3a where $\widehat{\mathcal {H}^j}$ is plotted. It is evident from the figure that there is a strong separation between the two populations. The Wilcoxon test (p-value = 1.8346e−36 and confidence interval [1.6942, 1.9675]), used because of the non-normal distribution of classes, confirmed the separation. Sample Entropy failed to separate the two classes, see Fig. 3b.

The receiver operating characteristic (ROC) curves of the two classifiers are shown in Fig. 3e, f. The Area Under Curve (AUC) for the $\widehat{\mathcal {H}}$-based LTC is $97.2\%$, while the AUC for the Sample Entropy-based classifier is $62\%$. The $\widehat{\mathcal {H}}$-based classifier ROC curve suggests that the best threshold for the separation of the two classes is $\theta =0.8754$.

For each patient we extracted the values of the Betti numbers: even if there are less epileptic than healthy signals with $\beta _0$ (3 vs. 12), this difference is not significant (p-value = 0.6946, Wilcoxon test). In Fig. 3h, i the generators of all the found *i*-dimensional holes, were grouped in a frequency histogram. We can recognise that the epileptic patients are characterised by 3 sensors (IDs 1, 2 and 5) while the healthy patients are characterised by sensors with IDs 1, 2, 3, 7, 10, 13 and 14. Those histograms are to be intended quantitatively: sensors involved in epilepsy spread are a few with respect to the ones involved in the normal brain activity.

## Limitations

The results for the classifier are very promising, even if we are aware that the reduced number of samples requires further investigations over the effectiveness of the method. Moreover, the role of generators should be deeply investigated. Nevertheless, we believe the present methodology provides a useful example regarding the use of TDA, especially in time series analysis.

## Abbreviations

AUC: Area Under Curve
EEG: electroencephalogram
H: persistent entropy
PCD: Point Cloud Data
ROC: receiving operative characteristic
TDA: topological data analysis
LTC: linear topological classifier
